# Comparison of embedded and added motor imagery training in patients after stroke: results of a randomised controlled pilot trial

**DOI:** 10.1186/1745-6215-13-11

**Published:** 2012-01-23

**Authors:** Corina Schuster, Jenny Butler, Brian Andrews, Udo Kischka, Thierry Ettlin

**Affiliations:** 1 Reha Rheinfelden, Salinenstrasse 98, 4310 Rheinfelden, Switzerland; 2 Faculty of Health and Life Sciences, Oxford Brookes University, Gipsy Lane, Oxford OX3 0BP, UK; 3 Nuffield Department of Surgical Sciences, John Radcliffe Hospital, Headley Way, Headington, Oxford, OX3 9DU, UK; 4 Brunel Institute for Bioengineering, Brunel University, Uxbridge, London, UB8 3PH, UK; 5 Oxford Centre for Enablement, Nuffield Orthopaedic Centre, Windmill Road, OX3 7HE, Oxford, UK; 6 Medical Faculty, University of Basel, Petersgraben 35, CH-4053 Basel, Switzerland

## Abstract

**Background:**

Motor imagery (MI) when combined with physiotherapy can offer functional benefits after stroke. Two MI integration strategies exist: added and embedded MI. Both approaches were compared when learning a complex motor task (MT): 'Going down, laying on the floor, and getting up again'.

**Methods:**

Outpatients after first stroke participated in a single-blinded, randomised controlled trial with MI embedded into physiotherapy (EG1), MI added to physiotherapy (EG2), and a control group (CG). All groups participated in six physiotherapy sessions. Primary study outcome was time (sec) to perform the motor task at pre and post-intervention. Secondary outcomes: level of help needed, stages of MT-completion, independence, balance, fear of falling (FOF), MI ability. Data were collected four times: twice during one week baseline phase (BL, T0), following the two week intervention (T1), after a two week follow-up (FU). Analysis of variance was performed.

**Results:**

Thirty nine outpatients were included (12 females, age: 63.4 ± 10 years; time since stroke: 3.5 ± 2 years; 29 with an ischemic event). All were able to complete the motor task using the standardised 7-step procedure and reduced FOF at T0, T1, and FU. Times to perform the MT at baseline were 44.2 ± 22s, 64.6 ± 50s, and 118.3 ± 93s for EG1 (N = 13), EG2 (N = 12), and CG (N = 14). All groups showed significant improvement in time to complete the MT (p < 0.001) and degree of help needed to perform the task: minimal assistance to supervision (CG) and independent performance (EG1+2). No between group differences were found. Only EG1 demonstrated changes in MI ability over time with the visual indicator increasing from T0 to T1 and decreasing from T1 to FU. The kinaesthetic indicator increased from T1 to FU. Patients indicated to value the MI training and continued using MI for other difficult-to-perform tasks.

**Conclusions:**

Embedded or added MI training combined with physiotherapy seem to be feasible and benefi-cial to learn the MT with emphasis on getting up independently. Based on their baseline level CG had the highest potential to improve outcomes. A patient study with 35 patients per group could give a conclusive answer of a superior MI integration strategy.

**Trial Registration:**

ClinicalTrials.gov: NCT00858910

## Background

Jean Decety (1996) defined motor imagery (MI) as a dynamic state during which a subject mentally simulates a given action without any motor output [1]. He further reviewed the neurophysiological basis of MI and suggested that both imagined and executed movements were found to activate similar regions of the premotor cortex, basal ganglia, and cerebellum that are associated with movement planning, execution, and modulation. Furthermore, an increase in heart rate, respiration frequency, and blood pressure were observed while imagining running, swimming, and weight lifting in healthy volunteers. In 1999 Jeannerod and Frak provided further evidence that the prefrontal cortex, pre-supplementary motor area (preSMA) and the parietal cortex might be involved in MI [2]. These neurophysiological findings have helped guiding the subsequent clinical introduction of MI in therapy.

At the beginning of the 21^st ^century attempts were made to transfer the concept of MI from sports psychology to stroke rehabilitation [3-6]. Page et al. and Liu et al. tried to combine occupational therapy and MI to improve motor recovery in patients after stroke or brain injury [3-10]. Page's concepts can be described as added MI. Patients after stroke in the subacute and chronic phase listened to a 10 minute pre-recorded tape with instructions to imagine movements that were previously practiced during therapy, e.g. weight-bearing and functional tasks. Movements were imagined from an external perspective in a visual mode three times per week over a four week period [3]. Subsequently, the simple MI intervention changed to a progressing procedure starting with a simple task, e.g. reaching for a cup, to more complex tasks, e.g. turning a book page [9]. Additionally, further MI training session elements changed over the years. MI perspective and MI mode changed to internal and kinaesthetic including imagination of sensations and feelings that were associated with the movement. MI training session duration increased from 10 to 20 minutes.

Liu et al. (2004) tested a more embedded MI approach during an occupational therapy intervention, rather than added MI, based on pictures showing tasks that have to be imagined over a two week period in patients with brain injury and stroke [7,8]. In this programme patients were also asked to imagine potential problems in performing the imagined task, to describe the problems verbally, to imagine the problem-solving version of the task, and, finally, to perform the corrected task physically after MI. MI training session were held one hour, three times per week. No information on MI mode and perspective were given.

Recently, embedded-focused MI interventions have become more popular. MI was not only applied after or during occupational therapy, MI was integrated into therapy routines in rehabilitation centres and nursing homes, in particular into physiotherapy, and speech and language therapy [11-13]. In a pilot study, Bovend'Eerdt and colleagues (2009) compared simultaneously performed MI versus muscle relaxation whilst manual stretches in patients with Multiple Sclerosis, brain injury, and after stroke [11]. In a further investigation, authors integrated MI into a six-week inpatient therapy setting with two to three MI training session per week [12]. MI was integrated in different kinds of therapy, e.g. physiotherapy and occupational therapy. Depending on the task to be imagined, MI was tailored to the patient needs. Both studies showed feasibility of MI trials during therapy sessions and the option to tailor MI content to patients with Multiple Sclerosis, after brain injury, and stroke. Braun et al. (2011) showed the practicability of the embedded MI integration approach in patients with Parkinson's disease [13]. A comparison of MI with muscle relaxation techniques during a six-week intervention period did not show significant differences but trends that patients of the MI group with milder disease showed a more improved walking performance than patients in a more severe disease stage.

To our knowledge, embedded and added MI approaches have not been compared and, therefore, it is unknown, which approach should be preferred in neurological rehabilitation. To address this, a randomised controlled pilot study comparing embedded and added MI was developed.

### Study aims

As suggested by Thabane et al. process and scientific aims were identified. In general terms, the process aims were to determine the feasibility and recruiting a sufficient number of subjects, who met the inclusion criteria and were able to perform the motor task [14]. The general scientific aims were:

1) to examine the feasibility of delivering the MI interventions;

2) to examine the efficacy of the MI interventions;

3) to examine the burden of the evaluation strategy;

4) to provide data to use for calculating the sample size necessary for a Phase III study.

Specific aims are provided in Table 1.

**Table 1:** Study aims and criteria of success

Aim category	Formulated aim	Study result
**Process aim**	a) To achieve an average patient recruitment rate of three patients per month.	Within 13 months of study duration 49 patients could be screened and 41 patients could be assessed and randomised. This corresponds to a recruitment rate of 3.2 to 3.8 patients per month.
	
	b) To be able to recruit patients with an ischemic or hemorrhagic stroke and to evaluate if patients' body weight limit up to 90 kg is manageable for assessors.	In total 29 patients with an ischemic and 10 patient with a hemorrhagic stroke participated in the study. One patient exceeded a body weight of 90 kg. Management of patients with this high body weight level depends on the motor function ability and therefore, on the level of help needed rather than on the weight itself.
	
	c) To be able to perform the motor task with 90% of all patients.	In total, 40 of 41 patients were able to perform the motor task at BL. At T0, T1, and FU all patients were able to perform the motor task.

**Scientific aim**	a) 90% of patients per group understand and perform the required MI intervention in the provided dosage and frequency.	All patients understood and performed the required MI intervention. One patient in EG1 could not perform the complete embedded MI intervention during the first and second session. Due to time constrains one patient in EG2 did receive only two of six intervention sessions.
	
	b) 90% of patients were able to perform all assessments in the given time frame and procedure for all measurement events.	The applied assessment procedure was feasible for all patients. The required time frame up to 3 hours at BL was tolerated due to short breaks.
	
	c) A sample size calculation could be performed based on the obtained assessments regarding time in seconds needed to perform the motor task.	Based on the collected data, a sample size calculation for a subsequent phase III trial and a post hoc power for the pilot study could be performed based on the primary outcome measure time needed to perform the motor task.

BL Baseline measurement event

FU Follow-up measurement event

EG1 Experimental group 1 (embedded MI)

EG2 Experimental group 2 (added MI)

MI Motor imagery

T0 Pre-intervention measurement event

T1 Post-intervention measurement event

## Methods

### Study design and blinding

**Figure 1:**
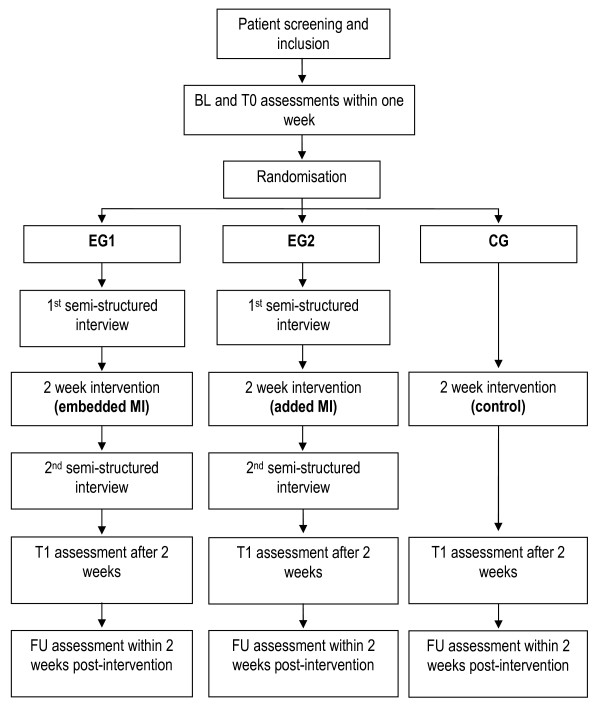
**Study overview**. BL Baseline measurement event, T0 Pre-intervention measurement event, T1 Post-intervention measurement event, FU Follow-up measurement event, EG1 Ex-perimental group 1, EG2 Experimental group 2, CG Control group.

The study was a mixed methods pilot RCT to prepare a subsequent phase III trial with three patient groups: two experimental (EG1, EG2) and one control group (CG). Semi-structured interviews were conducted with patients in EG1 and EG2 once before and after the MI intervention. The methodology of the qualitative part is described elsewhere [15]. Due to the extensive interview analyses results of the qualitative part will be described in a separate report. Figure 1 indicates the measurement events and study arms. All three groups received standardised physiotherapy treatment focussing on balance. Experimental groups received embedded (EG1) or added MI training (EG2), whereas CG listened to audio tapes with information related to stroke. All patient treatments were performed by one therapist not blinded to group allocation. Two blinded examiners performed all necessary assessments twice at baseline (BL), before intervention (T0), after intervention (T1), and after a two-week follow-up (FU) period. The study was implemented according to the Declaration of Helsinki and was approved by the ethics committee at the School of Health and Social Care at Oxford Brookes University, Oxford (UK) and the responsible Swiss ethics committee (Aarau, Kanton Aargau, Switzerland, reference number: 2008/077). The trial was registered with ClinicalTrials.gov: NCT00858910.

### Randomisation and allocation concealment

After giving written informed consent, patients underwent two measurement events (BL, T0) before randomisation. An independent researcher, who did not work in our institution, produced a computer-generated randomisation list (MATLAB 2007b, Mathworks Inc., USA) and sent it to the pharmacist in our institution. The pharmacist created sealed envelopes including group allocation, each for one patient. Before the second baseline assessment (T0) the project leader requested the sealed envelope respective to the patient number from the pharmacist and gave it to the patient after finalisation of T0. If possible, patients unsealed the envelope themselves. Both (researcher, pharmacist) were not involved in the current study. After patient randomisation allocation to the study groups (EG1, EG2, CG) documents were stored with patient's personal documents in a locked cabinet. Patients were verbally instructed not to discuss group allocation or therapy content until the post-intervention assessment has been performed. The independent examiner was unaware of the randomisation until the last follow-up assessment of the patient had been performed.

### Participants

Patients were recruited from the database of the rehabilitation centre, according to the inclusion criteria: first ischemic or hemorrhagic stroke at least 3 months before, able to stand with or without a cane for at least 30 seconds on a normal hard floor, able to walk 20 metres with or without a cane or an orthosis, older than 18 years, score at least 20 on the Mini-Mental State Examination, given written informed consent. Patients were excluded if they had: joint replacements (knee, hip, shoulder), motor task limiting pain in the upper or lower body evaluated with the 11-point visual analogue scale, limited range of motion in the hip, knee, ankle joints or toes, bodyweight exceeding 90 kilograms, or had a comprised mental capacity to give written informed consent.

### Study interventions

#### Motor task

**Figure 2:**
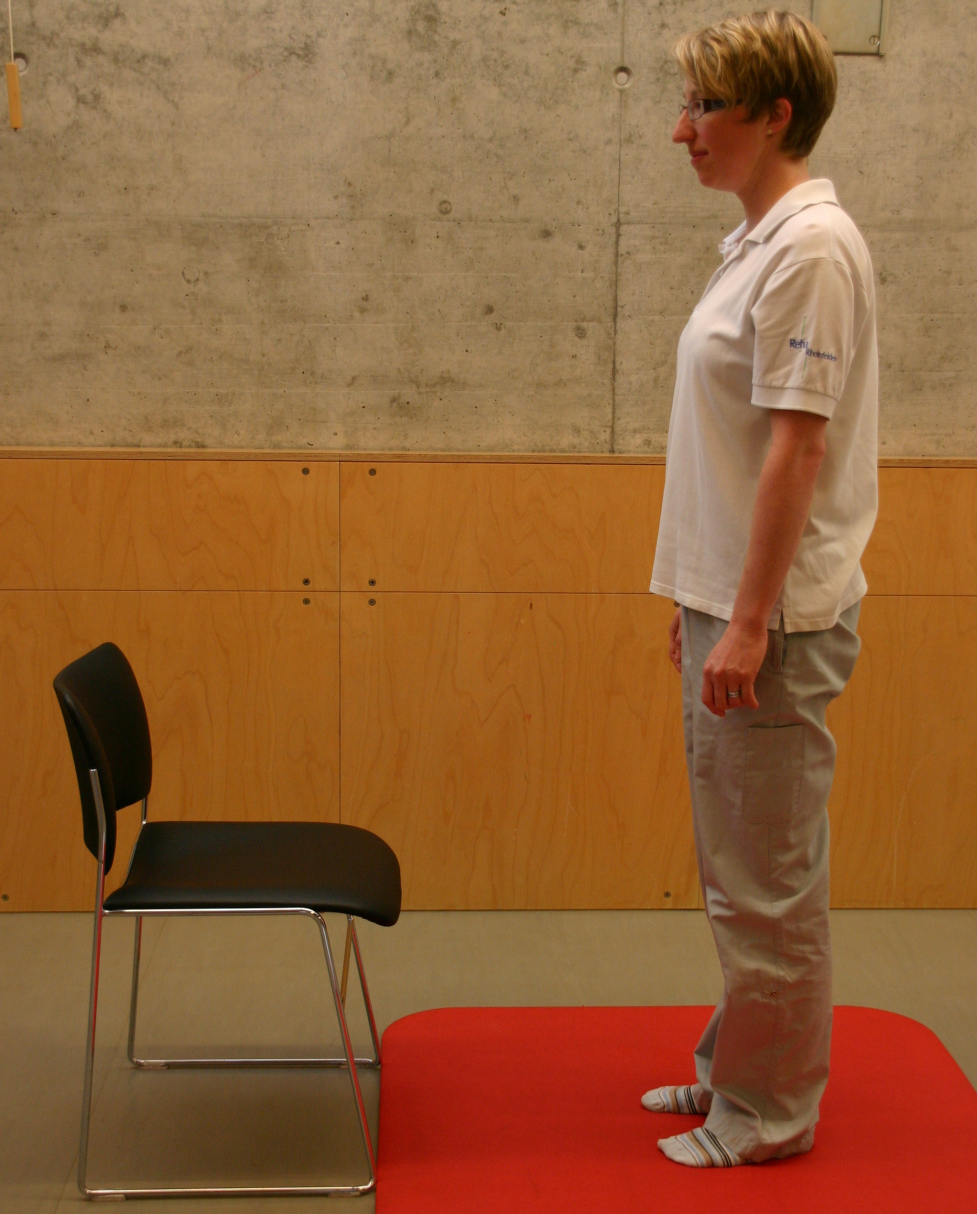
**Motor task stage 0: Standing**.

**Figure 3:**
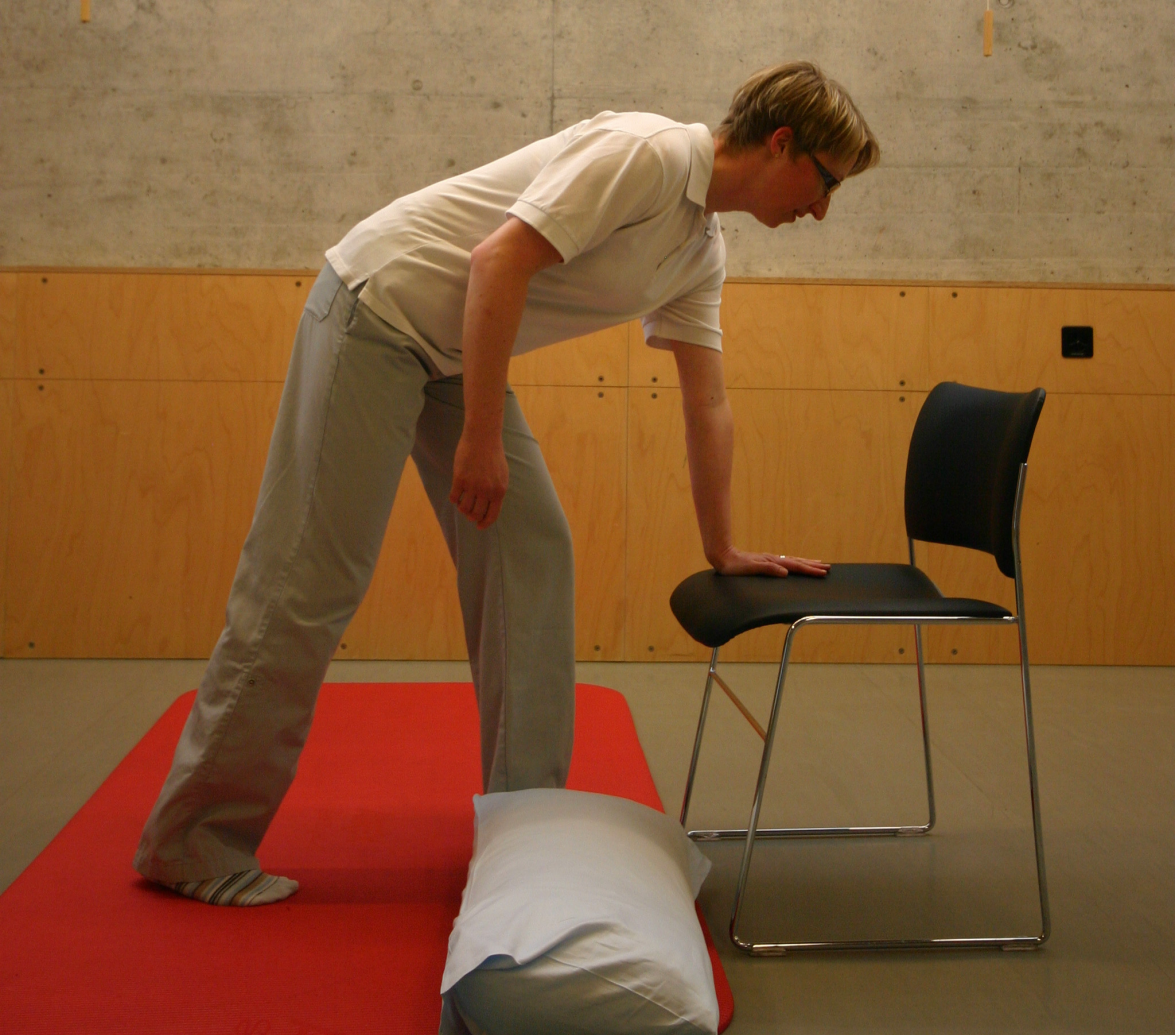
**Motor task stage 1: Stride standing**.

**Figure 4:**
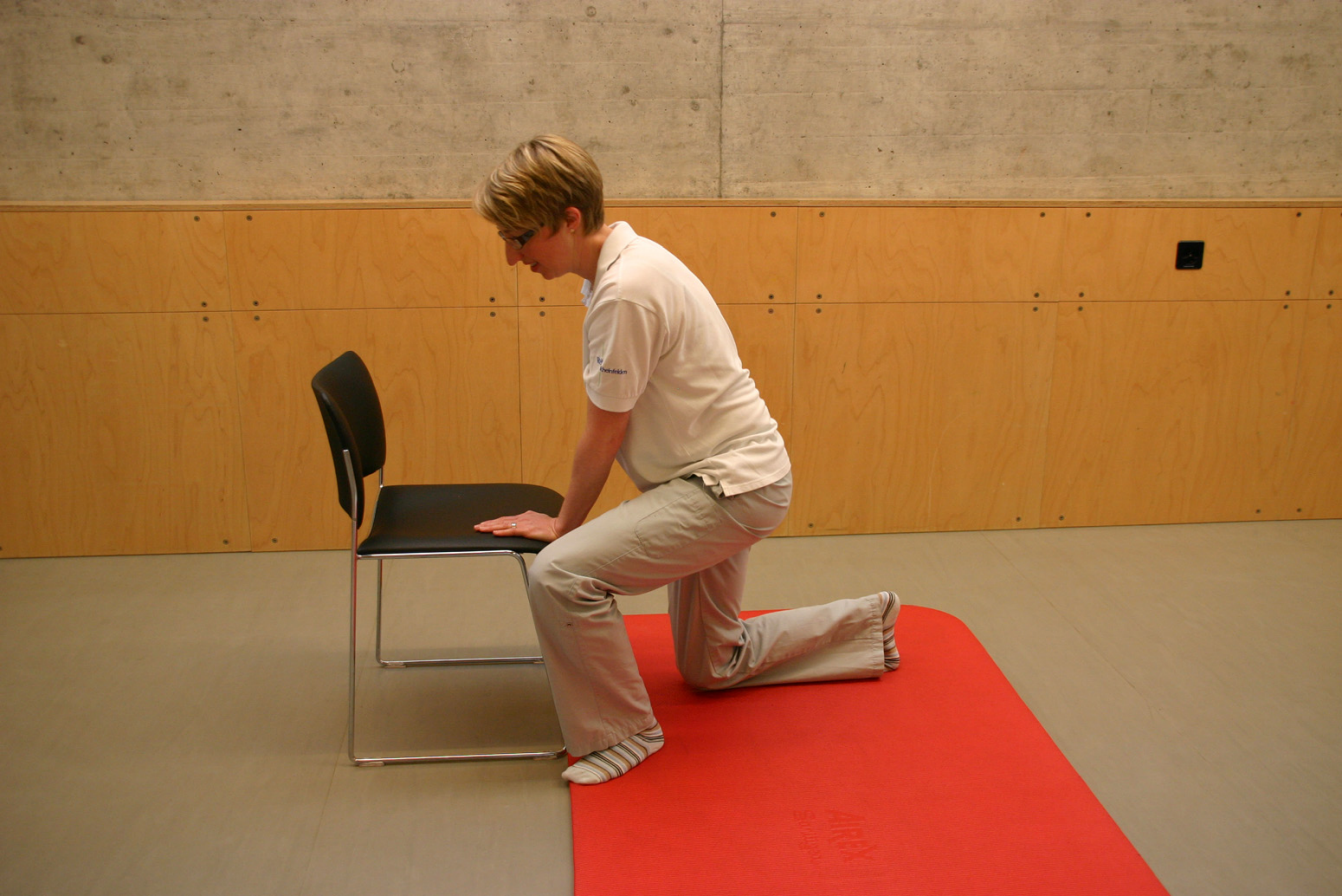
**Motor task stage 2: To half-kneeling**.

**Figure 5:**
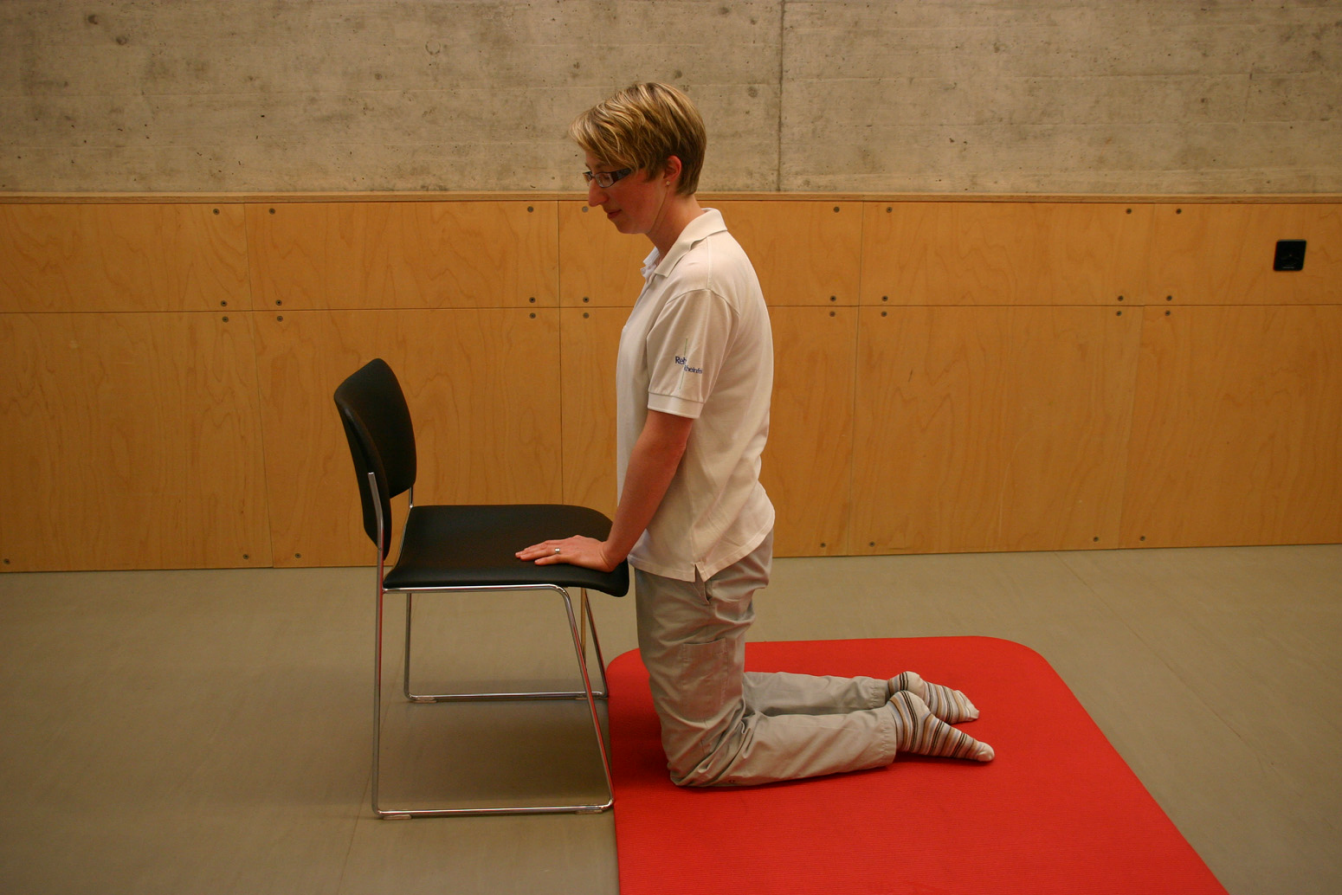
**Motor task stage 3: To high-kneeling**.

**Figure 6:**
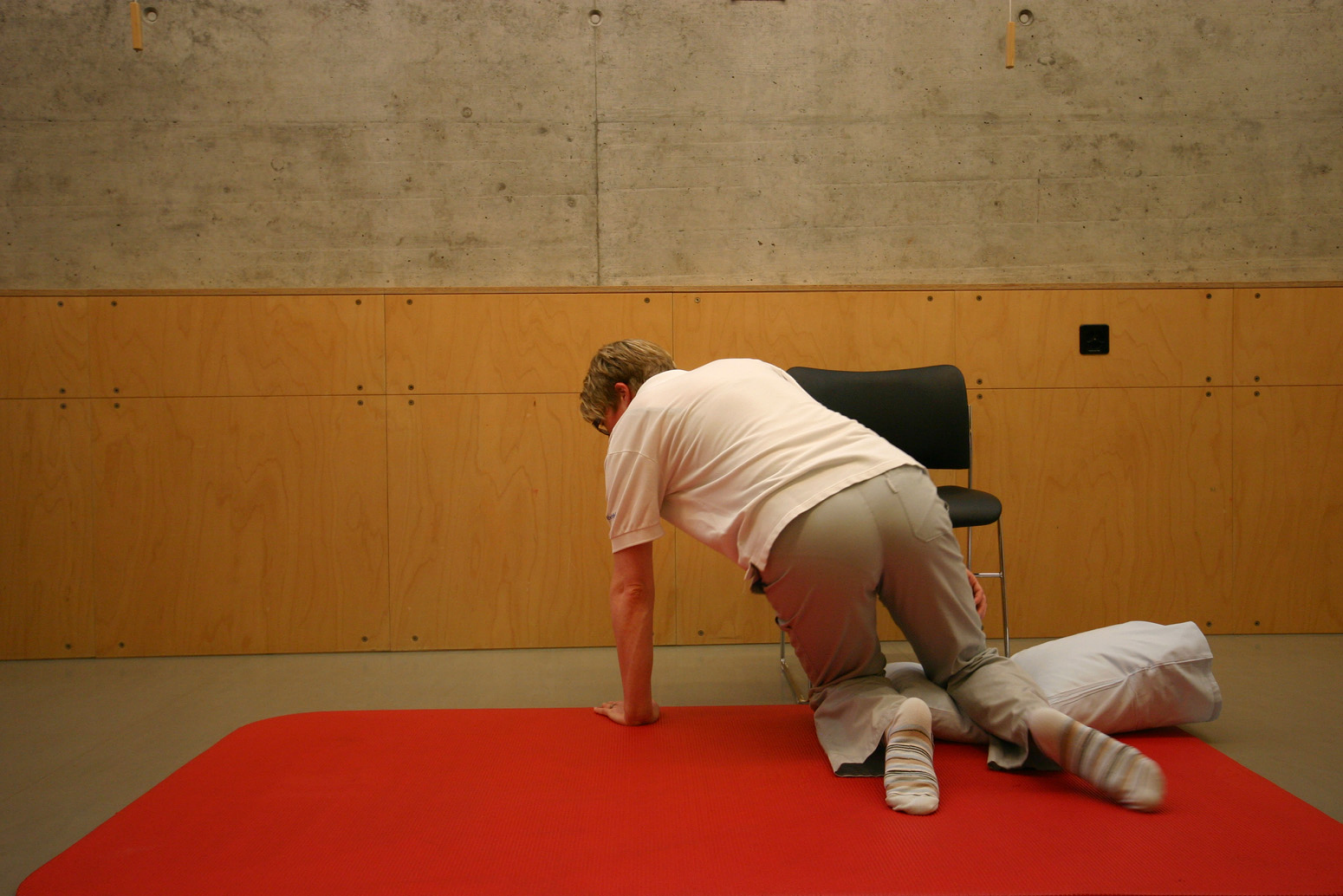
**Motor task stage 4: To half-sitting**.

**Figure 7:**
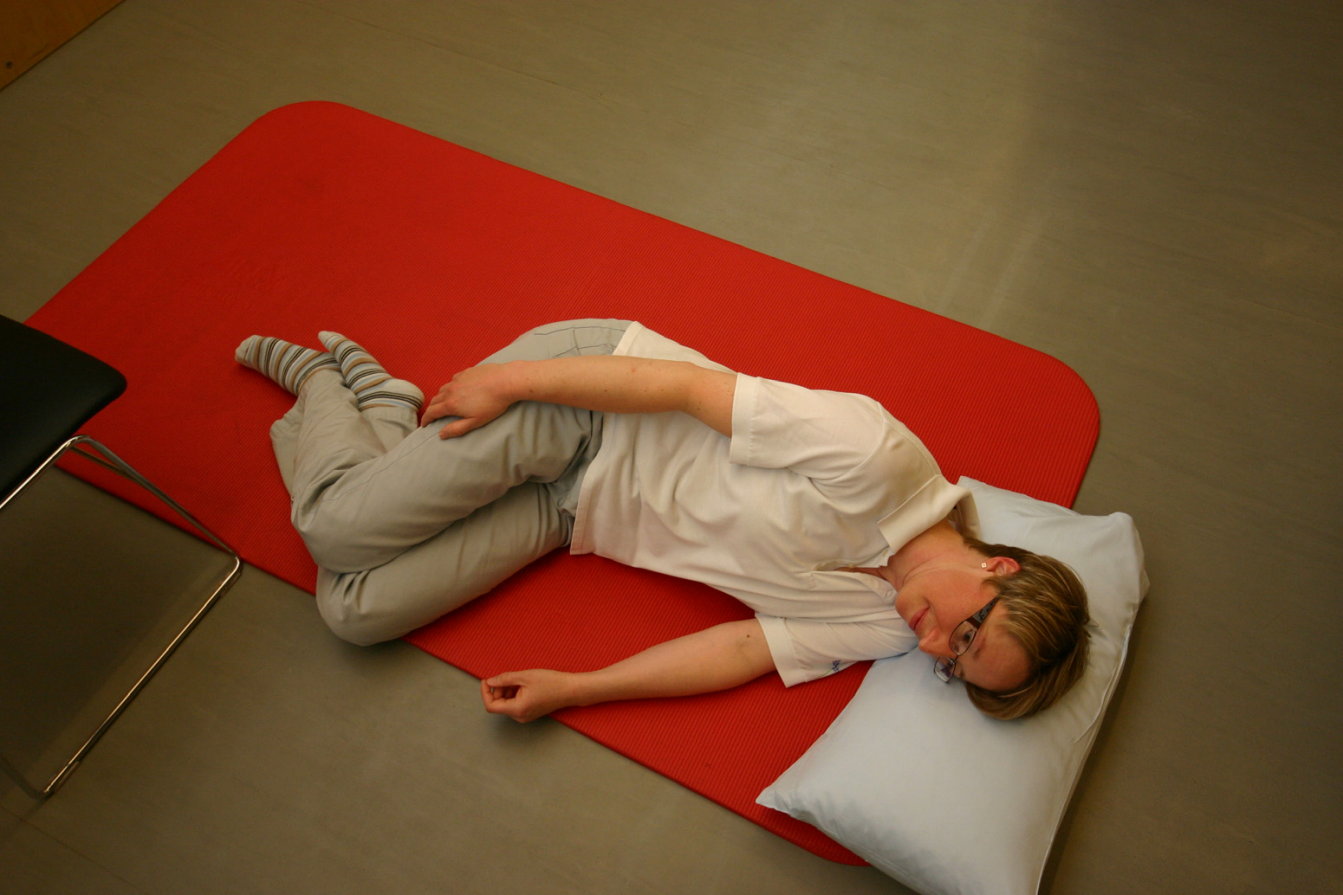
**Motor task stage 5: To side laying**.

All three study groups had to perform the motor task 'Going down, laying on the floor, and getting up again' ten times: during the four measurement events and in each of the six physiotherapy sessions. The motor task was modified from the task of Adams and Tyson (2000) [16]. Two of their proposed 13 stages of the task were modified: the starting position (stride standing) was included as the first stage because stride standing is already challenging for patients after stroke. The original stage 5 (to prone kneeling and up) was left out because only a small number of patients were able to maintain an upright posture with the affected upper limb while maintaining the unaffected arm in an extended position. All stages are described in Table 2 and shown in Figure 1, Figure 2, Figure 3, Figure 4, Figure 5, Figure 6, Figure 7, and 8. Patients progressed from one stage to the next without stand-ing up in between. After reaching the stage supine lying on a mat on the floor, patients rested for a short while, typically less than ten seconds, before getting up again in the reversed stage order. Patients could rest on the floor as long as they wished. To determine the time needed to perform the motor task from video recording, the resting time was excluded from the analyses. Materials used to perform the motor task included a chair without armrest, a red mat, two small and two large pillows for padding during the task if necessary and for the head, while lying on the side or supine on the mat. Patients were free in their selection of the foot that stood in front during stride standing, half-kneeling and high-kneeling on mat while lying down and getting up again.

**Table 2:** Description of motor task

Stage	Modified stages	Comment	Stage	Recommended stages	Illustration
0	Standing	Freely, no chair support	**0**	Standing	Please see Figure 2.

1	Stride standing	Non-affected hand rests on the chair without armrests	**1**	Stride standing, non-affected leg comes to front	Please see Figure 3.

2	To half-kneeling on to a large foam wedge	Non-affected hand rests on the chair without armrests			Not applicable

3	To half-kneeling on to a small wedge	Non-affected hand rests on the chair without armrests			Not applicable

4	To half-kneeling on a mat	Non-affected hand rests on the chair without armrests	**2**	To half-kneeling on knee of affected leg on a mat	Please see Figure 4.

5	To high-kneeling on a mat	Non-affected hand rests on the chair without armrests	**3**	To high-kneeling on a mat	Please see Figure 5.

6	To half-sitting on two pillows	Non-affected hand on mat			Not applicable

7	To half-sitting on one pillow	Non-affected hand on mat			Not applicable

8	To half-sitting on a mat	Non-affected hand on mat	**4**	To half-sitting on the non-affected side on a mat	Please see Figure 6.

9	To side lying on a large wedge	Laying on non-affected side, head padded on one small pillow			Not applicable

10	To side lying on a small wedge	Laying on non-affected side, head padded on one small pillow			Not applicable

11	To side lying on a mat	Laying on non-affected side, head padded on one small pillow	**5**	To side laying on a mat	Please see Figure 7.

12	To supine lying on a mat	Head padded on one small pillow	**6**	To supine laying on a mat	Please see Figure 8.

**Figure 8:**
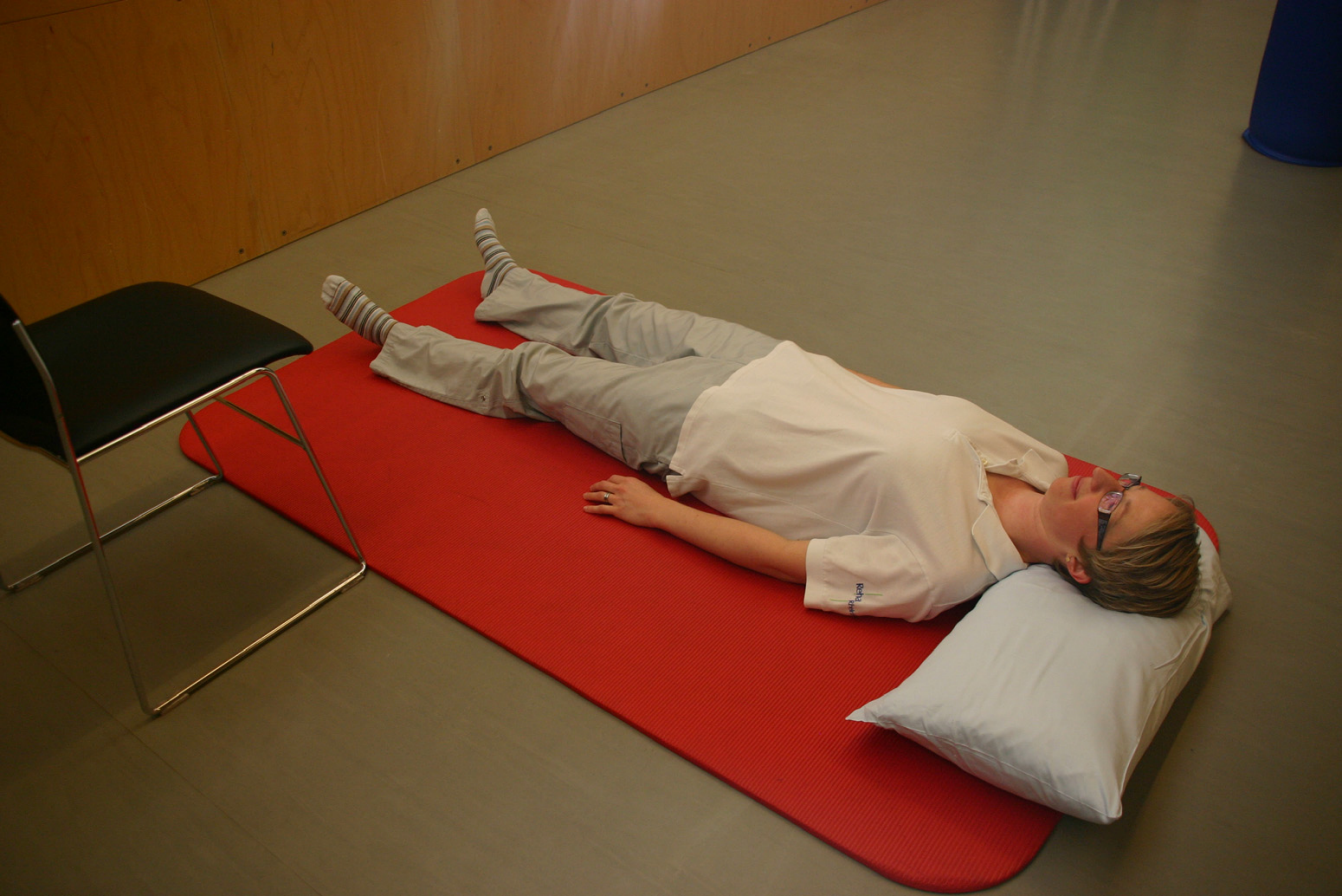
**Motor task stage 6: To supine laying**.

#### Physiotherapy

All patients received six physiotherapy sessions over the two week intervention period. The session content was based on a mixed neuro-physiological and motor learning approach [17]. Patients were treated by an experienced physiotherapist with twelve years of practice in neurological rehabilitation. Each session lasted 25 to 30 minutes. Depending on the motor level of the patient, the sessions included activities while lying, sitting, standing, and walking. The main content focused on exercises and activities to improve postural control in different starting positions, preferable positions (or surfaces) with small support to bear body weight (e.g. sitting, standing). The motor task 'Going down, laying on the floor, and getting up again' was practiced once during physiotherapy in all study groups. In the therapy sessions it was not allowed to practice the motor task more than once, in a different order, or parts of the motor task on a treatment bench. Patients were asked not to practice the motor task at home during the intervention period. To enable comparability all physiotherapy sessions were video-recorded.

#### Embedded (EG1) and added MI training (EG2)

Table 3 provides an overview of the MI training session elements for embedded and added MI. In EG1 the MI training was embedded into physiotherapy of the six therapy sessions based on the work of Liu et al. In total, treatment time was about 45 to 50 minutes [7,8]. Furthermore, suggestions from the PETTLEP framework published by Holmes et al. (2001) were considered [18]. The seven capital letters represent the following aspects of a MI intervention:

**Table 3:** Overview on training session elements for embedded and added MI

	MI training session element	MI training session elements for embedded MI	MI training session elements for added MI
**MITS elements**	Integration of MI (embedded, added)	**Embedded **into physiotherapy session	**Added **after physiotherapy session
	
	Session (group or individual)	Individual session	Individual session
	
	Temporal order	MI trials **before **physical practice trial	MI trials **after **physiotherapy session
	
	Supervision by an instructor	**Supervised**	**Not supervised**
	
	Directedness with stepwise guidance	Directed	Directed
	
	Location of MITS (task-specific, not task-specific)	**Task-specific: **during physiotherapy on red mat with chair for support	**Not task-specific: **after physiotherapy session in separate room on a treatment bench
	
	Position of the individual during MI (task-specific, not task-specific)	**Task-specific: **depending on the motor task stage that has to be imagined	**Not task-specific: **supine lying on a treatment bench
	
	Instruction medium (acoustic)	Spoken instructions **directly **from therapist	Spoken instructions from therapist **on tape**
	
	Instruction type (detailed, keywords, coarse)	Detailed	Detailed
	
	Instruction individualisation (standardised, tailored)	Standardised	Standardised
	
	Instruction mode (live, pre-recorded)	**Live**	**Pre-recorded**
	
	Eyes (open, closed)	Closed	Closed
	
	Perspective (internal, external)	Internal	Internal
	
	Mode (kinaesthetic, visual)	Both: first visual MI, then kinaesthetic MI	Both: first visual MI, then kinaesthetic MI
	
	Focus (motor, cognitive, strength)	Motor	Motor
	
	Familiarisation with MI before intervention start	None	None

**Temporal parameters**	Number of MI trials in one MITS	5 to 9 visual, 2 to 4 kinaesthetic	6 to 8 visual, 1 to 3 kinaesthetic
	
	Duration of one MITS	embedded into physiotherapy: 15 to 20 min	added after physiotherapy: 15 to 20 min
	
	Total MI time within 6 MITS	6× MI training session duration = 90 to 120 min	6× MI training session = 90 to 120 min

Words in bold indicate differences between embedded and added MI training sessions.

MI Motor imagery

**- Physical / Emotion: **Imagination of the motor task where it should be performed, without any prior relaxation exercises, in an active and alert state.

**- Timing: **Duration of the motor task should not exceed the real performance duration.

**- Environment: **Using (personalised) multisensory environmental cues.

**- Task / Learning / Perspective: **Patients, who preferred the external MI perspective, were asked to switch to the internal perspective after learning and familiarisation with the motor task.

The complete motor task was divided into its thirteen stages. Each stage was imagined five times before it was physically practiced once. At the end of each physiotherapy session, patients imagined the complete task four times while lying supine on the treatment bench and four times while standing against a wall. To control for every imagination trial each of the eight MI trials were timed with a stop watch by the patient and by the therapist.

In EG2 patients received about 30 minutes of physiotherapy in each session before they were offered an added MI training, which based on the studies of Page et al. [4,9,19]. Patients listened to a tape that consisted of three parts: part one was a brief relaxation period (about 3.5 minutes), afterwards in part two (14.5 minutes), patients listened to the description of each motor task stage that should be imagined, and were instructed to imagine the complete task as often as possible. Finally, in part three, patients had a short period to refocus on the room and the situation (two minutes). The total intervention time per session was about 45 to 50 minutes. Patients listened to the tape in a separated, quiet room in a padded supine lying position on a treatment bench.

#### Control group (CG)

Besides receiving physiotherapy during a 30 minutes session, participants in the CG listened to a 17 minutes tape (average). The total intervention time per session was about 45 to 50 minutes. The rationale for this was to provide CG participants the same therapeutic attention as applied in EG1 and EG2. The tape started with a short relaxation period (about 3.5 minutes). Afterwards patients listened to information about stroke: its cause, its consequences for different body functions and its recovery phase, therapy options, prevention of potential complications, self-help groups and their offers. This control protocol has been used in other MI studies without negative effect reported by authors [3,9]. Similar to EG2 the third part of the tape included a short period to refocus on the room and the situation (2 minutes). All tapes had an encouraging character and patients were asked how they liked the information on the tape. Patients listened to the tape in a separate, quiet room in a padded supine laying position on a treatment bench.

### Assessments used

The assessments used for the four different outcome profiles will be described briefly. A detailed description can be found in the published study protocol by Schuster et al. [15]. All assessments were used in their German version. The primary outcome is the time difference in seconds to perform the motor task from pre to post-intervention. It was obtained by the recorded video of the task performed. The following four profiles were assessed in all patients:

#### 1) The motor task related profile

included - the time difference in seconds between T1 and FU,

- patient's help needed to perform the motor task was evaluated with the seven classification levels of the Chedoke-McMaster Stroke Assessment activity scale (CMSA, 7 = independent performance without help or safety concerns, 1 = total assistance or the task is not tested for safety reasons) [20],

- achieved stage of the motor task based on a modified classification of Adams and Tyson [16] (please see 'Stages of the motor task'), and

- 'Imagination inflation' by patients' predicted time to perform the task at each measurement event.

#### 2) The motor impairment and balance profile

included - the extended Barthel index with 16 items, which evaluated patients' performance of activities of daily living on a five-point Likert scale with a total score of 64 (0 = cannot perform the task, 4 = independent) [21], and

- the Berg Balance Scale, which evaluated patients' performance in 14 balance task of different levels on a 5-point scale with a total score of 56 (0 = cannot perform the task, 4 = task fulfilled) [22].

#### 3) The motor imagery profile

included - the computer-based Imaprax questionnaire (version 1.1, 2001-2004). Patients were seated in front of a laptop to watch the Imaprax videos. The software itself was operated by the examiner. Six gestures or activities of daily living were evaluated in a standardised three step procedure: patients were asked 1) to select the correct gesture or activity from three proposed ones, 2) to evaluate the vividness of their 'inner picture', and 3) to determine the internal or external perspective used for their 'inner picture'. During step 2, patients were presented five videos showing the same person performing the same gesture but in different vividness levels. Additionally, patients were offered two options to rate their 'inner picture' as more or less vivid than in the watched videos. In total, vividness could be rated on a 7-point scale [23], and

- the kinaesthetic and visual imagery questionnaire (KVIQ), which was specifically developed to assess motor imagery ability for individuals with motor impairments [24]. The questionnaire is available in a short (10 items) and a long version (20 items). The latter version was used in this investigation, which includes all items of the short version. All items were evaluated while sitting in a standardised sequence for visual and kinaesthetic subscale: 1) the examiner showed the movement once, 2) the patient performed the just seen movement once from a standardised starting position, 3) the patient imagined the movement once from the internal perspective, and 4) the patient scored the vividness of the 'inner picture' on a 5 point Likert scale (1 = 'no image', 5 = 'image as clear as actually seeing it') as well as the feeling associated with the imagined movements (1 = 'no sensation', 5 = 'as intense as making the movement') [23].

#### 4) The psychological profile

included - the evaluation of patient's fear of falling using the Activities-Specific Balance Confidence Scale to assess patients' self-perceived confidence to remain balance in 16 different situations. The ques-tionnaire was completed during a face to face interview using a visual analogue scale (zero to 100 percent (10 cm) [25],

- the patients' intrinsic motivation evaluated from the patient's MI diary. Using details on frequency of independent MI practice reported in the patient's diary motivation to practice and the compliance with the training can be determined, and

- the patient's wellbeing enquired by a direct question: 'How do you feel today?'. This was scored on an 11-point visual analogue scale ranging from zero (very good) to ten (very bad).

Furthermore, patients' handedness and cognitive function were assessed with the Edinburgh Handedness Inventory and the Mini-Mental State Examination [26,27].

### Examiner experience

Patients were assessed by two examiners. Both were physiotherapists with more than ten years of working experience. One holds a Master's and the other a Bachelor's degree. The examiners were trained by the first author to become familiar with the test administration and patient handling. The training included three hours of direct instruction, twice assistance during patient testing and twice supervision during own test administration with patients. Regular meetings during the study implementation ensured consistency in test administration.

### Patient diary

Regardless of their group allocation all patients received a study diary. The aim was to note the date and time of the next therapy, the number of additionally practiced MI trials of the motor task and other practiced MI or physical tasks outside the therapy. Furthermore, patients had the opportunity to comment on things that went well or were problematic. Patients received their diary after the first therapy session and handed it back after the intervention at the T1 measurement event. They had to bring it to all sessions. The treating therapist asked at the beginning of each session if and what patients had practiced in between. If the patient was not able to write it down the therapist did so at the beginning of the session.

### Data analysis

**(a) Descriptive data **were calculated representing frequencies, means, and standard deviations for patient's personal, motor task, and different profile data.

**(b) Inter-rater reliability **was calculated for all objectively-assessed motor task related measures: time and help needed to perform motor task, and the Berg Balance Scale at BL, T0, T1, and FU. Intraclass correlation coefficients (ICCs) were calculated with the two-way mixed model (ICC(3,1)) and 95% confidence intervals (CI).

**(c) **Measured **assessment data **at BL and T0 were calculated with $\left(\frac{BL+T0}{2}\right)=PRE$ to estimate one pre-intervention score. Data were analysed with the help of an 'intention-to-treat' analysis.

**(d) Missing values **for continuous scaled variable were determined by calculating the mean change of the variable from PRE to T1 or from T1 to FU, respectively. The estimated mean value was added or subtracted to/from the last available measured value. This procedure was applied on four patients for KVIQ and Imaprax values (at PRE, T1, or FU). Missing values for nominal scaled variables were determined by using the 'last available value carried forward' method. This procedure was applied in three patients for foot position during the motor task in the phase of going down.

**(e) **Continuous variables were tested for **normal distribution **and **variance homogeneity **to test for independent T-test and ANOVA requirements.

**(f) Baseline differences **of three study groups were tested with Student's independent T-test or Kruskal-Wallis test in case of no normal data distribution, which occurred for the variable number of falls since stroke onset and scores of the Activities-Specific Balance Confidence Scale in EG1. P-values for group comparisons were given in Tables 4 and 5.

**(g) **To test the effect of MI for time needed to perform the motor task as dependent variable, a two-factor **ANOVA **was applied with independent variables group (EG1, EG2, CG) as between-subjects factor and time (PRE, T1, FU) as within-subject factor [28]. If compound symmetry was lacking, a correction according to Greenhouse-Geisser (epsilon correction) was employed [28,29].

**(h) **For all further profile assessments the Kruskal-Wallis test was applied to compare related means of three groups. Paired T-tests were computed to determine significant changes from PRE to T1 and from T1 to FU for each group. Except for the Berg Balance Scale, due to the lack of normal distribution, the Friedman test and the Wilcoxon signed-rank test was applied. The 'imagination inflation' (ImaIn) effect was determined for two measurement events: T0 and T1. The following ratio was calculated: ImaIn $=\frac{t_{estimated}}{t_{recoreded}}$ (t = time needed to perform the motor task). This ratio was group-wise compared with independent T-tests to check for differences between experimental groups, and experimental groups and control group.

**(i) **Partial eta squared (η**^2^**) is reported for estimation of the effect size [29]. The calculated partial η**^2 ^**was used to compute an a priory **sample size **for an appropriate powered subsequent RCT with Gpower 3.0 [30].

**(j) Frequency analysis **was used to determine practice intensity and intrinsic motivation based on the patients' diary entries.

All analyses were performed with the Statistical Package for Social Sciences version 16, 2007 (SPSS, Inc., Chicago Ill) with p ≥ 0.05.

## Results

### Descriptive information (process aim)

**Figure 9:**
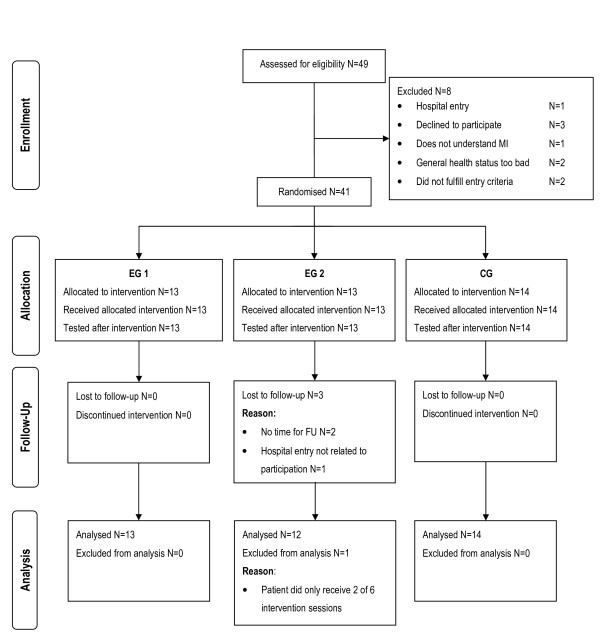
**Patient analysis flow chart**. BL Baseline measurement event, T0 Pre-intervention measurement event, T1 Post-intervention measurement event, FU Follow-up measurement event, EG1 Experimental group 1, EG2 Experimental group 2, CG Control group, N Sample size.

The study was conducted in a mid-sized rehabilitation centre in the North-Western part of Switzerland. Participants were recruited between 1^st ^April 2009 and 31^st ^May 2010. Figure 9 represents the patients study flow chart. After T0 41 patients were randomised resulting in an allocation of 13 patients to EG1 and CG, and 14 patients to EG2. Table 4 provides an overview on participants' descriptive information and baseline comparability. Patients were not comparable in all baseline characteristics. Patients in CG experienced significantly more falls since stroke onset and needed more time to perform the motor task than both experimental groups. Unexpectedly, 29 of 39 included patients scored above 45 points in the Berg Balance Scale including 14 patients with a scoring of 55 and 56 points, respectively.

**Table 4:** Patient descriptive data and group equality at PRE

Group	EG1 N = 13	EG2 N = 12	CG N = 14	p-value at BL
Age	65.8 ± 10.2	59.7 ± 13.0	64.4 ± 6.8	0.20

Gender (females)	3	5	4	N/A

Weight (kg)	73.8 ± 10.9	76.8 ± 9.0	75.9 ± 10.4	0.47

Years of education	11.5 ± 2.4	13.5 ± 3.1	12.8 ± 4.1	0.089^#^

Marital status (married)	11	8	12	N/A

Diagnosis (CVA)	11	9	9	N/A

Handedness before stroke (right)	10	11	14	N/A

Affected body side (right)	9	7	6	N/A

Time since stroke onset (years)	2.9 ± 1.9	4.3 ± 3.6	3.5 ± 3.9	0.22

MMSE (PRE, 30)*	25.0 ± 2.3	27.5 ± 2.2	27.2 ± 1.6	0.006^2^

EBI (PRE, 64)*	60.7 ± 4.5	61.2 ± 2.3	59.7 ± 5.1	0.35^1^

Falls since stroke onset	0.4 ± 0.7	1.6 ± 1.0	2.1 ± 1.3	< 0.001^#^

Walking aid^§^	1	5	5	N/A

Orthosis (AFO)	1	5	5	N/A

Numbers are listed as frequency or mean score ± 1 standard deviation.

AFO Ankle foot orthosis to prevent foot drop during walking

CG Control group

CVA Cerebrovascular accident

EBI Extended Barthel Index

EG1 Experimental group 1 (embedded MI)

EG2 Experimental group 2 (added MI)

Kg Kilogramme

MMSE Mini-Mental State Examination

N Sample size

N/A Not applicable

PRE Pre-intervention score

* Maximal score

^§ ^Walking aid includes any kind of stick or rollator walker

# Variable was not normally distributed at PRE

^1 ^No equality of variances in independent T-test EG1 and EG2

^2 ^Group differences between EG1 and CG, not between EG2 and CG

### Assessment duration and inter-rater reliability (scientific aim)

Depending on the amount of information and assessments to administer the duration varied among the four measurement events. On average, assessment duration at BL lasted 2.5 hours, at T0 1.5 hours, at T1 1.5 hours and at FU 1 hour. Both assessors were tested on data of a pilot patient, whose data were not included in the main analyses and on the first eight included participants. At BL inter-rater reliability for time to go down and up was 0.99 (0.97 < × < 0.99), for help to go down and up was 0.93 (0.75 < × < 0.98), for the Berg Balance Scale 0.92 (0.68 < × < 0.98). Calculations were repeated for T0, T1, and FU and remained on the high level (between 0.91 and 1.00).

### Primary outcome (scientific aim)

**Figure 10:**
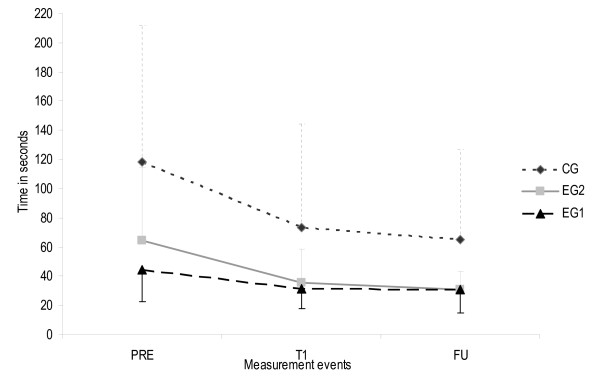
**Time needed to perform the motor task for all measurement events**. Error bars show one standard deviation of the means. The upper limit for CG and EG2, and the lower limit for the EG1 were added to remain easy and fast readability of the figures. EG1 Experimental group 1, EG2 Experimental group 2, CG Control group, PRE Pre-intervention (scores from BL and T0 were calculated with $\left(\frac{BL+T0}{2}\right)=PRE$ to estimate one pre-intervention score), T1 Post-intervention (after 2 week intervention period), FU Follow-up (2 weeks after intervention finalisation).

ANOVA revealed a statistically significant effect of the factor time. All three groups could perform the motor task faster after the two week intervention period (F(2, 36) = 19.14, p < 0.001, η^2 ^= 0.35, observed power = 0.995), which remained after the two week follow-up period (F(2, 36) = 4.77, p = 0.036, η^2 ^= 0.12, observed power = 0.565). Baseline equality and homogeneity of variances were not given for time needed to perform the motor task. Therefore, the Greenhouse-Geisser correction was applied [28]. Figure 10 displays time needed to perform the motor task for all measurement events. ANOVA revealed no effect for factor group (F(2, 36) = 1.55, p = 0.199, η^2 ^= 0.079, observed power = 0.454).

### Secondary outcomes (process and scientific aims)

#### 1) Motor task related profile

**Figure 11:**
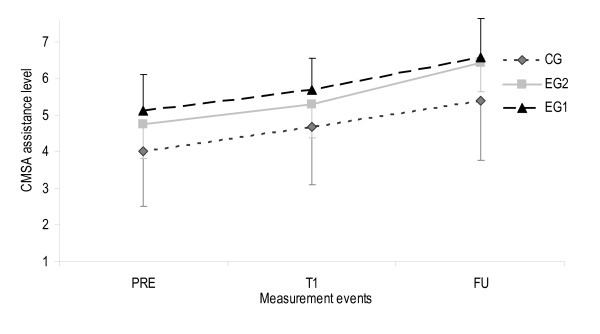
**Help needed to perform the motor task for all measurement events**. Error bars show one standard deviation of the means. The upper limit for CG and the lower limit for EG1 and EG2 were added to remain easy and fast readability of the figures., EG1 Experimental group 1, EG2 Experimental group 2, CG Control group, PRE Pre-intervention (scores from BL and T0 were calculated with $\left(\frac{BL+T0}{2}\right)=PRE$ to estimate one pre-intervention score), T1 Post-intervention (after 2 week intervention period), FU Follow-up (2 weeks after intervention finalisation).

**Help needed to perform the motor task: **A second two-factor ANOVA analysis was applied to estimate the effect of MI regarding help needed. Homogeneity of variances was not employed for help needed at T1 and FU. The Greenhouse-Geisser correction determined a clear improvement for all groups from PRE to T1 and T1 to FU (F(2, 36) = 77.37, p < 0.001, η^2 ^= 0.68, observed power = 1.0 and F(2,36) = 42.71, p < 0.001, η^2 ^= 0.54, observed power = 1.0). Figure 11 provides an overview on all measurement events.

**Motor task stages: **At BL, T0, T1 and FU all patients performed all stages of the motor task except for one female, who stopped at stage 1 at BL because of a high level of fear of falling.

**Use of pillows: **At BL 31 of 39 patients did not need any pillows, at T0 33, at T1 36, and at FU 36. One pillow was used in seven patients at BL, in four at T0, in three patients at T1 and FU. In total, two patients needed two pillows at BL and T0 but only one during the post-intervention assessment events T1 and FU. The pillow for the head is not counted in the analysis.

**Foot positioning: **Patients were offered to put their non-affected leg in front during stride standing to go down (stage 1) but they were not restricted to this leg. In total, 32 patients put their non-affected leg in front and kneeled on their affected leg at BL, 29 at T0, 32 at T1, and 29 patients at FU.

To get up again and move from high kneeling to half-kneeling on a mat (from stage 5 to stage 4) 29 patients positioned their non-affected foot in front at BL, 31 at T0, 27 at T1, and 26 at FU. From stage 1 to stage 0, 26 patients moved their affected leg forward to their non-affected leg at BL, nine patients moved their non-affected leg forward, two patients moved their affected leg backwards, and two patients moved their non-affected leg backwards. The frequency of these analyses remained almost the same until FU.

#### 2) Motor imagery ability profile

**Figure 12:**
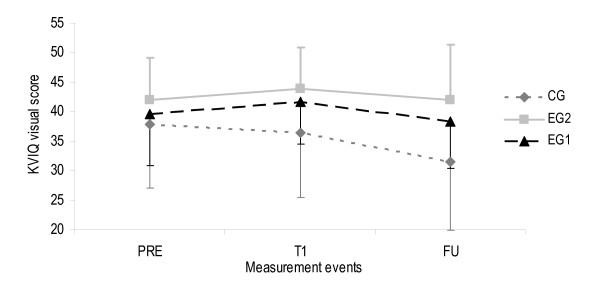
**Visual subscale values of the KVIQ for all measurement events**. Error bars show one stan-dard deviation of the means. The upper limit for CG and EG2, and the lower limit for the EG1 were added to remain easy and fast readability of the figures. KVIQ Kinaesthetic and visual imagery questionnaire (scoring range between 20 and 50), EG1 Experimental group 1, EG2 Experimental group 2, CG Control group, PRE Pre-intervention (scores from BL and T0 were calculated with $\left(\frac{BL+T0}{2}\right)=PRE$ to estimate one pre-intervention score), T1 Post-intervention (after 2 week intervention period), FU Follow-up (2 weeks after intervention finalisation).

**Figure 13:**
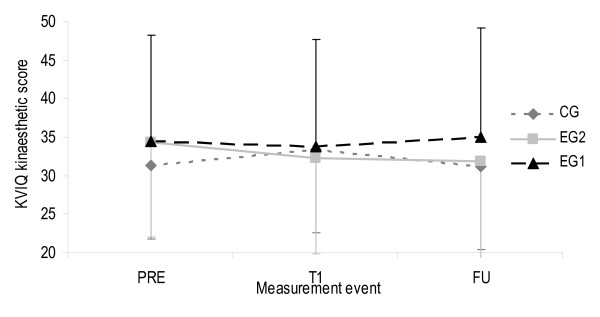
**Kinaesthetic subscale values of the KVIQ for all measurement events**. Error bars show one standard deviation of the means. The upper limit for CG and the lower limit for EG1 and EG2 were added to remain easy and fast readability of the figures. KVIQ Kinaesthetic and visual imagery questionnaire (scoring range between 20 and 50). EG1 Experimental group 1, EG2 Experimental group 2, CG Control group, PRE Pre-intervention (scores from BL and T0 were calculated with $\left(\frac{BL+T0}{2}\right)=PRE$ to estimate one pre-intervention score), T1 Post-intervention (after 2 week intervention period), FU Follow-up (2 weeks after intervention finalisation).

A third two-factor ANOVA analysis was performed for variable imagery ability to evaluate MI intervention effect on patients' MI ability. Normal distribution and homogeneity of variances were redeemed. For the visual subscale ANOVA revealed a significant change between PRE and T1 but not between T1 and FU (F(2,36) = 5.58, p = 0.006, η^2 ^= 0.13, observed power = 0.84). For the kinaesthetic subscale no significant changes were observed. No group interactions were determined for both subscales. Figures 12 and 13 provide an overview on both KVIQ subscales and all measurement events. The visual subscale showed a slight increase from PRE to T1 and a decrease from T1 to FU in EG1 and EG2. CG showed a scoring decrease from PRE to T1 continuing to FU. The kinaesthetic subscale shows contradictory scoring development for both, EG1 versus EG2 and CG.

#### 3) Further assessments

Scoring of all further profiles at PRE, T1 and FU and respective p-values are displayed in Table 5. No significant changes were detected. Tests for 'Imagination inflation' for the primary outcome time needed to perform the motor task revealed no significant effect between embedded and added MI, and between experimental groups and CG.

**Table 5:** Changes in profile-specific assessments

Group	Experimental group 1 N = 13			Experimental group 2 N = 12			Control group N = 14		
	
	PRE	Change PRE to T1	Change T1 to FU	PRE	Change PRE to T1	Change T1 to FU	PRE	Change PRE to T1	Change T1 to FU
Imaprax: visual (42)*	32.6 ± 3.8	0.6 ± 3.7	0.6 ± 2.4^§^	32.1 ± 4.6	1.5 ± 1.8	-0.3 ± 1.8^§^	31.5 ± 4.0	-0.4 ± 4.8	-1.0 ± 3.0^§^

BBS (56)*	52.5 ± 5.2	0.3 ± 1.7	0.2 ± 1.1	50.8 ± 4.0	1.0 ± 2.7	0.02 ± 2.5	46.9 ± 9.3	1.9 ± 3.0	0.2 ± 2.0

ABC-Scale (100)*	80.5 ± 20.4	0.9 ± 8.7	2.3 ± 6.2	72.4 ± 20.0	3.8 ± 6.1	4.5 ± 10.1	70.4 ± 22.5	3.0 ± 10.3	3.0 ± 9.0

Wellbeing (VAS, 10)*	2.6 ± 1.6	-0.1 ± 2.0	-0.7 ± 1.6	2.4 ± 1.3	0.1 ± 1.8	0.7 ± 1.2	2.1 ± 1.5	0.2 ± 1.7	-0.3 ± 1.6

Numbers are listed as mean score ± 1 standard deviation.

ABC Activities-Specific Balance Confidence Scale

BBS Berg Balance Scale

FU Follow-up

PRE Pre-intervention score (mean score from BL and T0)

T1 Post-intervention score

VAS Visual analogue scale

* Maximal score

^§ ^p < 0.001

### Therapy analyses (scientific aim)

In total, 227 out of 234 planned therapies for all groups were applied. Seven were not utilized due to patients' time constraints. In EG1 one therapy was not administered, in EG2 four, and in CG two thera-pies. Overall, 35 patients received six therapies, three patients five and two patients received four therapies. Wellbeing was rated high for all groups and for all six therapy sessions: EG1 from 1.9 ± 1.7 to 2.5 ± 2.5, EG2 remained at 2.8 ± 1.4, CG from 2.2 ± 1.4 to 2.0 ± 2.4. Temporal parameters for MI are displayed in Table 3.

### Patient diary (scientific aim)

Diary usage varied among patients. The most commonly named reasons for not filling in the diary or no MI or PP apart from therapies were time constraints or inability to fill in. In rare cases, the diary was left at home. All diaries were handed back after intervention for analyses. On average, two patients in CG, nine patients in EG1, and five patients in EG2 practiced per day, including days with and without therapy sessions. Patients in CG practiced toning/tensioning or breathing exercises from the tape they had listened to or tasks in different starting positions that were practiced in the therapy sessions. Patients in EG1 and EG2 imagined the motor task mainly during sitting but also during standing tasks on different times of the day. In total, patients in CG practiced a task once a day. In EG1 and EG2 patients imagined the motor task 2.5 times and 1.2 times per day, respectively.

### Type II error and sample size calculation (scientific aim)

The results of the ANOVA of the primary outcome suggest accepting of the null hypothesis (H_0_), implying that there is no difference between both experimental groups [28]. Based on the small sample size this could lead to a type II error (β). To positively influence the occurrence of a type II error, it is suggested to loosen the significance level or to increase the investigated sample size [28]. For the primary outcome measure the ANOVA results determined p = 0.199. To loosen the α-level to p ≥ 0.10 would have no effect on determining a group difference between EG1 and EG2. Therefore, an a priori sample size for a future trial was calculated based on the results of the actual investigation. Gpower was used to calculate an effect size of 0.316 based on the partial η^2 ^of 0.091, which can be seen as medium effect [30]. The sample size calculation was performed using the following parameters: F-test, ANOVA repeated measures, within-between interaction, effect size F = 0.316, α = 0.05, power = 0.8, number of groups = 2, measurement repetitions = 3, correlations among repeated measures = 0, nonsphericity correction ε = 0.6. Gpower revealed a total sample size of 48. With a drop out rate of 20%, loss to follow-up and uncertainty in sample size calculation, it is suggested to include 33 patients per group in a future study to compare embedded and added MI if time needed to perform the motor task will be chosen as the primary outcome measure. A 0.48 post hoc power for the current pilot study was calculated with Gpower using the computed effect size 0.316 (α = 0.05, sample size = 39, numerator = 1, number of groups = 3). A total of at least 80 patients should have participated in the current investigation to achieve a power of 0.8.

## Discussion

The pilot study tested the feasibility of supplementing MI training to physiotherapy. Specifically, MI training was embedded into physiotherapy and added after physiotherapy to learn a complex motor task: 'Going down, laying on the floor, and getting up again'. Furthermore, both MI integration approaches were compared to a control group that listened to tapes with information on stroke. All further factors regarding the study interventions remained the same for all groups. All groups received the same amount of attention and kind of physiotherapy content. They showed significant changes in the primary outcome measure time needed to perform the motor task from pre to post-intervention. The significant improvement could be maintained during follow-up period, which is an important aspect of therapy intervention studies [31]. No group differences in time needed to perform the motor task was detected from pre to post-intervention.

Patients in all groups showed a high compliance and were highly motivated. Frequently named reasons for study participation were to help other patients after stroke with the research findings. Furthermore, patients were interested to learn the MI technique. All were able to learn the task, completed all 13 stages, and were able to improve the motor task performance regarding time and help needed considering the long time period and functional level since stroke onset and study participation.

### Different MI integration approaches

Both MI interventions were designed based on currently accepted MI intervention paradigms. Embedded MI based on the work from Liu et al. and the PETTLEP framework from sports psychology [7,18], whereas added MI was derived from the results of Page's publications [4,5]. In a recently published systematic literature review on motor imagery elements the authors described 17 MI training session elements [32]. Embedded MI (EG1) and added MI (EG2) differed in seven MI training session elements: integration, temporal order, supervision, location, position of the individual, instruction medium and instruction mode (for more details please refer to Table 3). Nevertheless, the current investigation suggests that the design differences have no influence on the effect of MI to learn the complex motor task. The same review analysed 129 MI interventions with positive changes in the pre to post-intervention assessments regarding their temporal parameters, suggesting an average MI training session duration of 17 minutes. Furthermore, we hypothesise that a MI intervention duration longer than two weeks including more MI training session is more important than the duration of one single MI training session. This hypothesis is supported by the results of the review mentioned above [32].

As suggested by Driskell et al. (1994), it is important to maintain patients' motivation for a positive overall effect of MI [33]. In our study, some patients in EG2 mentioned that listening to the same tape became less interesting after the fourth time. On the other hand, patients in EG1, in particular patients ≥ 80 years of age, mentioned the difficulty to capture all details and motor task order to imagine during the first two sessions. Both occurrences showed that duration and content play an important role to learn and further use MI independently. Therefore, we suggest implementation of a modified content to be imagined, especially if the motor task to be imagined includes whole body movements more than focusing on one limb only, e.g. make a step with one leg to stand in stride standing.

### The motor task

To the authors' knowledge, the motor task 'Going down, laying on the floor, and getting up again' was investigated in stroke patients for the first time. The motor task was modified after the work from Adams and Tyson (2000) [16]. At T0 all patients were able to perform the complete motor task using a chair with no armrests and a thin mat. Pillows were only needed to pad 1) the head while side and supine laying, 2) knees due to temporal pain caused by degenerative joint diseases, and 3) arches of the feet and toes due to a temporally muscle tension increase or stretching of the muscles. All named reasons can be associated to the patients' age and the time period between stroke onset and study entry. As carried out in the current investigation, the motor task did not cause any harm to the patients. On the contrary, in combination with the applied physiotherapy the practiced motor task contributed to a decrease of fear of falling assessed by the Activities-Specific Balance Confidence Scale. The motor task seems to be feasible and practicable to be learned and performed by stroke patients. Therefore, for further motor task practice, we recommend using only seven of the 13 stages listed in Table 2. For both motor task related assessments as well as time and help needed, all raters showed a high inter-rater reliability. Furthermore, scoring the help needed to perform the motor task using the independence levels of the CMSA activity subscale was reasonable. The lower the assistance a patient required (higher CMSA level) the closer was her/his performance to healthy individuals [20]. As expected, patients' level of help needed changed over time and was adapted to the actual situation according to the CMSA guidelines. Primarily, help was needed if the patients did not know how to proceed to the next stage of the motor task or if the therapists had safety concerns. We did not expect that the help provided reduced the time needed to perform the motor task compared to an independent motor task performance.

### Motor imagery ability

Scoring for the visual and kinaesthetic subscales at PRE are comparable with published data of stroke patients by Malouin and colleagues in 2007 [24]. All three groups started almost at the same visual MI ability level. As expected, both MI integration approaches helped to improve patients' visual MI ability from PRE to T1. In general, kinaesthetic values were lower than visual values but patients in CG scored lowest at PRE. At T1 both experimental groups decreased, whereas CG increased the scoring. At FU EG2 and CG decreased the kinaesthetic scoring almost to the same value but EG1 increased the MI ability to a higher level than at PRE. We hypothesise that those patients in EG1 and EG2 learned to clearly distinguish between visual and kinaesthetic MI during the investigation. Therefore, they were able to show the difference in the scoring at T1 and FU. Contrary, not all patients in CG were able to differentiate to the same amount as in EG1 and EG2. This indicates that patients might have to be asked at all measurement events if they can differentiate between visual and kinaesthetic MI. The application of the Imaprax software before administering the KVIQ clearly helped to determine the patients' preferred MI perspective. It serves as basis for the use of the first person perspective during the KVIQ. Overall, patients in EG1 were able to improve their kinaesthetic MI ability at FU, whereas patients in EG2 got worse.

### Sample size

The decision to extend the study sample up to 15 patients per group was based on two reasons: Firstly, based on previous therapy intervention studies in our clinic a high drop out rate was expected. Secondly, MI interventions based on previous motor imagery studies published by Page et al. (2001) and Liu et al. (2004) [4,7] reported high effect sizes. Unfortunately, other researchers conducting MI intervention studies at the same time as the current pilot study reported no effect of their motor imagery interventions [11-13,34]. To not to underestimate or overestimate the effect of MI the pilot study sample has been raised to obtain more detailed data providing sufficient information for a subsequent Phase III study.

### Study limitations

Based on the classification by Thabane et al. the pilot study outcome can be classified as feasible with modifications [14]. Results of the current investigation have to be interpreted with caution due to the following limitations: Firstly, the sample size in all three groups was too small consequently increasing the risk of a type II error. Secondly, notwithstanding the randomised group allocation, patients in the three study groups were not comparable in all baseline characteristics. Though randomly allocated, patients in CG experienced significantly more falls since stroke onset and needed more time to perform the motor task than both experimental groups. Furthermore, CG showed the lowest scoring in the Berg Balance Scale and the Activities-Specific Balance Confidence Scale. Therefore, CG had the highest potential to improve their outcomes, in particular, their motor task performance. Due to the small sample size for each group statistical analyses corrected for baseline imbalances would not have been appropriate. A motor impairment assessment, e.g. the CMSA, would have added a better description of the patients' functional status at study entry. This has been omitted due to the already long duration of up to three hours of the measurement events. Thirdly, the motor task including whole body movements might have been too complex for stroke patients to imagine. Published successful MI investigations had chosen single limb or bimanual movements, e.g. turning a page, grasping a cup, and hang out laundry [5,7]. Klausler (1991, cited in Jarus, 2000) pointed out that older adults pay more attention to irrelevant task details or could have problems with the information organisation [31]. Therefore, we propose to cut a complex motor task that involves the whole body into shorter pieces to be imagined and give the patient the opportunity to add piece after piece to a consolidated motor task part for forward and backward chaining.

Finally, the MI assessments Imaprax and KVIQ at BL and T0 were used as familiarisation sessions to learn how MI works and can be used. More effort should be undertaken to prepare the patient for a MI intervention, e.g. make sure that patients know the difference between visual and kinaesthetic imagery and can distinguish between internal and external MI perspective.

### Recommendations for further MI investigations

An appropriate sample size of a comparison of embedded and added MI would be 33 per group if time needed to perform the motor task (continuous data level) would be chosen as primary outcome measure (see section 'Type II error and sample size calculation' above). If help needed to perform the motor task would be chosen as primary outcome measure (ordinal data level) a much larger sample size would be required suggesting a multicentre study design. We suggest replacing the Berg Balance Scale with the CMSA to perform a group allocation based on stratified randomisation to correct for imbalances in patients' motor function. Regardless their motor function level, patients were well adapted to maintain balance in different positions and situations assessed with the Berg Balance Scale. Patients with a low motor function level achieved a Berg Balance Scale scoring above 45 points, which is an indication that they are safe in independent walking despite their low motor function level [35]. Furthermore, a detailed MI ability assessment and MI familiarisation sessions should be administered to enable the patient to know important MI training session elements, e.g. distinguishing between visual and kinaesthetic MI modes and an internal or external MI perspective. For both MI integration approaches it is proposed to include a progression of the content if a complex motor task will be investigated. A clear description of the implemented MI training session elements and temporal parameters would be helpful to interpret study results within available literature.

## Conclusion

Embedded and added MI were demonstrated to be feasible and practicable for clinical implementation within a two week course of outpatient physiotherapy. This research has provided rigorous data for sample size calculations for further projects in this area of investigations. MI seems to be an abstract con-struct for patients after stroke, therefore, they should have a guided training prior implementing a MI intervention. Information should be given concerning important aspects of MI training session elements, e.g. distinguishing between visual and kinaesthetic MI modes and an internal or external MI perspective. It is suspected that patients need more time to learn a complex motor task with embedded MI. However, embedded MI enables patients after stroke to use and improve their MI ability, in particular to use kinaesthetic MI. For MI of complex motor tasks, a progression of the MI intervention is suggested with task segmentation and forward and backward chaining to the complete motor task MI visualisation.

The modified motor task was successfully performed by all participating patients after stroke. The standardized order helped the patients to reduce both the help needed to perform the motor task and their fear of falling. Therefore, the motor task 'Going down, laying on the floor, and getting up again', consisting of seven stages, should be included in physiotherapy sessions and practiced with all patients during every stage of the rehabilitation process on a regular basis.

## List of abbreviations

BL: Baseline; CG: Control Group; EG1: Experimental group 1 (embedded MI); EG2: Experimental group 2 (added MI); FU: Follow-up; KVIQ: Kinaesthetic and imagery questionnaire; MI: Motor imagery; PETTLEP: Physical; Environment; Task; Timing; Learning; Emotion; Perspective; PRE: Mean score of baseline (BL) and pre-intervention measurement event (T0); T0: Pre-intervention measurement event; T1: Post-intervention measurement event.

## Competing interests

The authors declare that they have no competing interests.

## Authors' contributions

CSch made substantial contributions to conception and design, acquisition of data, analysis and interpretation, and wrote the manuscript. JB, BA, UK, ThE made substantial contributions to conception and design, data analysis and interpretation, were involved in drafting the manuscript and revising it critically for important intellectual content. All authors gave final approval of the version to be published.

## References

[B1] Decety J The neurophysiological basis of motor imagery Behav Brain Res 1996 77 1-2 45 52 10.1016/0166-4328(95)00225-1 8762158

[B2] Jeannerod M Frak V Mental imaging of motor activity in humans Curr Opin Neurobiol 1999 9 6 735 739 10.1016/S0959-4388(99)00038-0 10607647

[B3] Page SJ Imagery improves upper extremity motor function in chronic stroke patients: a pilot study Occup Ther J Res 2000 20 3 200 215

[B4] Page SJ Levine P Sisto S Johnston MV A randomized efficacy and feasibility study of imagery in acute stroke Clin Rehabil 2001 15 3 233 240 10.1191/026921501672063235 11386392

[B5] Page SJ Levine P Sisto SA Johnston MV Mental practice combined with physical practice for upper-limb motor deficit in subacute stroke Phys Ther 2001 81 8 1455 1462 11509075 10.1093/ptj/81.8.1455

[B6] Page SJ Mental practice: a promising restorative technique in stroke rehabilitation Top Stroke Rehabil 2001 8 3 54 63 10.1310/7WDU-2P4U-V2EA-76F8 14523738

[B7] Liu KP Chan CC Lee TM HuiChan CW Mental imagery for promoting relearning for people after stroke: A randomized controlled trial Arch Phys Med Rehabil 2004 85 9 1403 1408 10.1016/j.apmr.2003.12.035 15375808

[B8] Liu KP Chan CC Lee TM Hui-Chan CW Mental imagery for relearning of people after brain injury Brain Inj 2004 18 11 1163 1172 10.1080/02699050410001671883 15545212

[B9] Page SJ Levine P Leonard A Mental practice in chronic stroke - Results of a randomized, placebo-controlled trial Stroke 2007 38 4 1293 1297 10.1161/01.STR.0000260205.67348.2b 17332444

[B10] Page SJ Hewett T Ford K Levine P Mental practice improves reaching kinematics in stroke Neurorehabil Neural Repair 2005 19 4 366

[B11] Bovend'Eerdt TJH Dawes H Sackley C Izadi H Wade DT Mental techniques during manual stretching in spasticity - a pilot randomized controlled trial Clin Rehabil 2009 23 2 137 145 10.1177/0269215508097298 19164401

[B12] Bovend'Eerdt TJ Dawes H Sackley C Izadi H Wade DT An integrated motor imagery program to improve functional task performance in neurorehabilitation: a single-blind randomized controlled trial Arch Phys Med Rehabil 2010 91 6 939 946 10.1016/j.apmr.2010.03.008 20510987

[B13] Braun S Beurskens A Kleynen M Schols J Wade D Rehabilitation with mental practice has similar effects on mobility as rehabilitation with relaxation in people with Parkinson's disease: a multicentre randomised trial J Physiother 2011 57 1 27 34 10.1016/S1836-9553(11)70004-2 21402327

[B14] Thabane L Ma J Chu R Cheng J Ismaila A Rios LP Robson R Thabane M Giangregorio L Goldsmith CH A tutorial on pilot studies: the what, why and how BMC Med Res Methodol 2010 10 1 10.1186/1471-2288-10-1 20053272 PMC2824145

[B15] Schuster C Butler J Andrews B Kischka U Ettlin T Comparison of embedded and added motor imagery training in patients after stroke: study protocol of a randomised controlled pilot trial using a mixed methods approach Trials 2009 10 1 97 10.1186/1745-6215-10-97 19849835 PMC2775030

[B16] Adams JMG Tyson S The Effectiveness of Physiotherapy to Enable an Elderly Person to Get up from the Floor: A single case study Physiotherapy 2000 86 4 185 189 10.1016/S0031-9406(05)60962-5

[B17] Pollock A Baer G Langhorne P Pomeroy V Physiotherapy treatment approaches for the recovery of postural control and lower limb function following stroke: a systematic review Clin Rehabil 2007 21 5 395 410 10.1177/0269215507073438 17613560

[B18] Holmes PS Collins DJ The PETTLEP approach to motor imagery: A functional equivalence model for sport psychologists J Appl Sport Psychol 2001 13 1 60 83

[B19] Page SJ Levine P Leonard AC Effects of mental practice on affected limb use and function in chronic stroke Arch Phys Med Rehabil 2005 86 3 399 402 10.1016/j.apmr.2004.10.002 15759218

[B20] Gowland C VanHullenaar S Torresin W Moreland J Vanspall B Barreca S Ward M Huijbregts M Stratford P Barclay-Goddard R Chedoke-McMaster Stroke Assessment: development, validation, and administration manual 1995 Hamilton (ON): School of Rehabilitation Science, McMaster University

[B21] Prosiegel M Böttger S Schenk T König N Marolf M Vaney C Garner C Yassouridis A Der Erweiterte Barthel-Index (EBI) - eine neue Skala zur Erfassung von Fähigkeitsstörungen bei neurologischen Patienten Neurol Rehabil 1996 7 7 13

[B22] Scherfer E Bohls C Freiberger E Heise KF Hogan D Berg-Balance-Scale - German Version - Translation of a Standardized Instrument for the Assessment of Balance and Risk of Fallingphysioscience 2006 2 59 66 10.1055/s-2006-926833

[B23] Schuster C Lussi A Wirth B Ettlin T Two assessments to evaluate imagery ability: translation, validity and test re-test reliability of the German KVIQ and Imaprax BMC Med Res Methodol 2011 under review 10.1186/1471-2288-12-127 PMC3528454 22905778

[B24] Malouin F Richards C Jackson P Lafleur M Durand A Doyon J The Kinesthetic and Visual Imagery Questionnaire (KVIQ) for Assessing Motor Imagery in Persons with Physical Disabilities: A Reliability and Construct Validity Study J Neurol Phys Ther 2007 31 1 20 29 17419886 10.1097/01.npt.0000260567.24122.64

[B25] Schott N German adaptation of the "Activities-specific Balance Confidence (ABC) scale" for the assessment of falls-related self-efficacy Z Gerontol Geriatr 2008 41 6 475 485 10.1007/s00391-007-0504-9 18327692

[B26] Oldfield RC The assessment and analysis of handedness: the Edinburgh inventory Neuropsychol 1971 9 1 97 113 10.1016/0028-3932(71)90067-4 5146491

[B27] Folstein MF Folstein SE McHugh PR "Mini-mental state". A practical method for grading the cognitive state of patients for the clinician J Psychiatr Res 1975 12 3 189 198 10.1016/0022-3956(75)90026-6 1202204

[B28] Munro BH Statistical methods for health care research 2005 5 Philadelphia, Penn.; London: Lippincott Williams & Wilkins

[B29] Pierce CA Block RA Aguinis H Cautionary note on reporting eta-squared values from multifactor ANOVA designs Educ Psychol Meas 2004 64 6 916 924 10.1177/0013164404264848

[B30] Faul F Erdfelder E Lang A-G Buchner A GPower 2007 3.0.8 Düsseldorf: Heinrich Heine Universität

[B31] Jarus T Ratzon NZ Can you imagine? The effect of mental practice on the acquisition and retention of a motor skill as a function of age Occup ther J Res 2000 20 3 163 178

[B32] Schuster C Hilfiker R Amft O Scheidhauer A Andrews B Butler J Kischka U Ettlin T Best practice for motor imagery: A systematic literature review on motor imagery training elements in five different disciplines BMC Med 2011 9 75 10.1186/1741-7015-9-75 21682867 PMC3141540

[B33] Driskell JC Copper C Moran A Does mental practice enhance performance? J Appl Psychol 1994 79 481 192

[B34] Braun SM Beurskens AJ Kleynen M Oudelaar B Schols JM Wade DT A Multicenter Randomized Controlled Trial to Compare Subacute 'Treatment as Usual' With and Without Mental Practice Among Persons With Stroke in Dutch Nursing Homes J Am Med Dir Assoc 2010 10.1016/j.jamda.2010.07.009 21450196

[B35] Berg KO Wood-Dauphinee SL Williams JI Maki B Measuring balance in the elderly: validation of an instrument Can J Public Health 1992 83 Suppl 2 S7 11 1468055

